# Clinical characteristics and recurrence predictors of sinonasal inverted papilloma: a retrospective cohort study

**DOI:** 10.3389/fonc.2025.1537905

**Published:** 2025-04-07

**Authors:** XiangHua Li, DongFei Wang, DeTao Yin

**Affiliations:** First Affiliated Hospital of Zhengzhou University, Zhengzhou, China

**Keywords:** sinonasal inverted papilloma, nasal endoscopic surgery, recurrence, smoking, previous surgery

## Abstract

**Objective:**

To investigate the clinical characteristics and predictors of postoperative recurrence in sinonasal inverted papilloma (SNIP).

**Methods:**

A retrospective cohort study of 53 SNIP patients treated at Zhengzhou University Hospital (2019–2023) was conducted. Patients were stratified into primary (n=34) and recurrent (n=19) cohorts. Clinical variables were analyzed using univariate and multivariate logistic regression.

**Results:**

Smoking (OR=10.08, 95% CI=1.32–77.15), prior surgery (OR=17.26, 95% CI=2.69–110.76), and ipsilateral involvement (OR=7.79, 95% CI=1.05–57.99) emerged as independent recurrence predictors (P<0.05). Krouse staging (T3 vs. T1–T2) showed no significant association (P=0.198).

**Conclusion:**

Comprehensive evaluation of smoking history, surgical history, and lesion laterality is critical for recurrence risk stratification in SNIP management.

## 1 Introduction

Sinonasal inverted papilloma (SNIP) accounts for 0.4-4.7% of sinonasal tumors, with an annual incidence of 0.74–1.5 cases per 100,000 population (1). Although histologically benign, SNIP exhibits locally aggressive behavior, high recurrence rates (15–20%), and malignant transformation potential (2). Patients typically present with unilateral nasal obstruction, headache, and rhinorrhea. Imaging features on computed tomography (CT), including characteristic bony destruction or hyperostosis, frequently involve the lateral nasal wall and maxillary sinus (3). Surgical resection remains the primary therapeutic approach; however, anatomical complexity and the tumor’s insidious growth pattern often lead to incomplete excision, resulting in postoperative recurrence (4, 5). Emerging evidence implicates smoking and human papillomavirus (HPV) infection in SNIP pathogenesis (6), though definitive etiological mechanisms remain elusive, complicating clinical management.

The clinical challenges of SNIP lie in its propensity for recurrence and malignant progression. While preoperative CT evaluation is routinely employed to delineate tumor extent and osseous involvement (7), its predictive value for recurrence risk remains underexplored. Environmental factors and chronic inflammatory stimuli have also been postulated to drive SNIP progression (8). Current studies on recurrence predictors remain fragmented, lacking systematic integration of tumor biology, imaging characteristics, and prognostic outcomes.

This study aims to identify independent predictors of SNIP recurrence through retrospective cohort analysis. By synthesizing clinical features (e.g., tumor location, staging) and surgical parameters (e.g., surgical approach), we propose to develop a risk stratification model to guide individualized treatment strategies. This investigation will not only inform surgical optimization but also establish an evidence-based framework for postoperative surveillance and preventive interventions (e.g., smoking cessation), ultimately reducing recurrence rates and mitigating malignant transformation risks.

## 2 Materials and methods

### 2.1 Study design and patient selection

A retrospective cohort study was conducted on 53 consecutive patients diagnosed with SNIP between January 2019 and January 2023 at the Otolaryngology Department of the First Affiliated Hospital of Zhengzhou University. Inclusion criteria required:1.Histopathologically confirmed SNIP diagnosis.2.Complete clinical and imaging records.3.Definitive surgical intervention with postoperative follow-up ≥12 months. Exclusion criteria included:1.Incomplete clinical data or lost-to-follow-up cases.2.Concurrent sinonasal malignancies.3.Non-surgical management.

Demographic variables (age, sex), comorbidities (allergic rhinitis, chronic diseases), behavioral factors (smoking, alcohol use), and tumor characteristics (laterality, Krouse staging, anatomical localization) were systematically extracted from electronic medical records (**Table 1**). Ethical approval was obtained from the Institutional Review Board of the First Affiliated Hospital of Zhengzhou University (Approval No.:2024-KY-1681-002).

**Table 1:** Demographic and clinical characteristics of patients (N=53).

Category	n (%)	Category	n (%)
Sex		Primary Anatomical Site	
Male	34 (64.2)	Nasal cavity	3 (5.7)
Female	19 (35.8)	Maxillary sinus	26 (49.1)
Age(yeas)		Ethmoid sinus	12 (22.6)
18-60	41 (77.4)	Frontal sinus	3 (5.7)
≥60	12 (22.6)	Pansinus involvement	8 (15.1)
Symptom Duration (Months)		Presenting Symptoms	
<12	27 (50.9)	Nasal obstruction	36 (67.9)
12–60	21 (39.6)	Headache	10 (18.9)
>60	5 (9.4)	Other symptoms*	7 (13.2)
Affected Nasal Cavity		Allergic Rhinitis Comorbidity	
left side	23 (43.4)	Yes	27 (50.9)
Offside	30 (56.6)	No	18 (34.0)
Comorbidities		Undetermined	8 (15.1)
Present	17 (32.1)	Krouse Staging System	
Absent	36 (67.9)	T1	3 (5.7)
Smoking History		T2	22 (41.5)
Yes	23 (43.4)	T3	28 (52.8)
No	30 (56.6)	Lesion Origin Site	
Surgical Approach		nasal cavity	2 (3.8)
Endoscopic surgery	40 (75.5)	Ostiomeatal complex	24 (45.3)
Combined approach	13 (24.5)	Other sites†	26 (49.1)

*Other symptoms included facial pain and rhinorrhea.

†Including sphenoid sinus and multiple-site involvement.

### 2.2 Surgical protocols

All procedures were performed under endoscopic guidance:1.Transnasal endoscopic approach via the middle meatus(n=40)2.Combined endoscopic and pre-lacrimal recess approaches(n=10) 3.Hybrid technique integrating brow incision with endoscopic access (n=3).

Surgical resection adhered to radical excision principles, including identification of tumor origin, mucosal stripping at the attachment site, and bone drilling or low-temperature plasma ablation to eradicate residual disease.

### 2.3 Observational factors

Clinical data included in univariate analysis were age, gender, duration of illness, smoking, whether allergic rhinitis was present, other chronic diseases, past surgeries, surgery type, lateralization of the lesion, tumor staging, and the localization of the tumor root. Factors from the univariate analysis that were associated with tumor recurrence(*P* < 0.05) were further included in multivariate analysis.

### 2.4 Statistical methods

Continuous variables were expressed as mean ± SD (Shapiro-Wilk normality test) and compared using Student’s t-test. Categorical variables underwent χ² or Fisher’s exact tests. Variables with *P* < 0.05 in univariate analysis entered multivariate logistic regression. Significance threshold: P<0.05 (SPSS 27.0).

## 3 Results

### 3.1 Clinical presentation

The cohort comprised 53 patients with sinonasal inverted papilloma (SNIP), including 34 primary cases (64.2%) and 19 recurrences (35.8%). Demographic characteristics revealed male predominance (64.2%, M:F=1.79:1), mean age 48.4 ± 13.9 years (range 23-81), and symptom duration 18.4 ± 29.8 months. All lesions were unilateral, with 96.2% (51/53) demonstrating endoscopically identifiable nasal cavity involvement. Primary clinical manifestations included nasal obstruction (67.9%), headache (18.9%), and purulent discharge (13.2%). Anatomical distribution predominated in the maxillary (49.1%) and ethmoid sinuses (22.6%), predominantly originating from the ostiomeatal complex (45.3%) and maxillary sinus walls.

### 3.2 Imaging findings

CT imaging revealed unilateral soft-tissue masses with characteristic papillary projections, predominantly involving the maxillary sinus medial wall and ostiomeatal complex. MRI demonstrated pathognomonic serpentine morphology on T2-weighted sequences, exhibiting cerebral gyri-like patterns.

### 3.3 Univariate analysis of factors influencing recurrence post-endoscopic surgery

The results indicated (see **Table 2**) that smoking, past surgical history, involvement of the right nasal cavity, and the location of the tumor root were associated with the recurrence of inverted papilloma (*P* < 0.05).

**Table 2:** Univariate analysis of factors associated with recurrence of sinonasal inverted papilloma.

Variable	All (n=53)	Primary cases (n=34)	Recurrent Cases (n=19)	Statistical Test (χ²/t)	*P-value*
Age (years), mean ± SD	48.4 ± 13.9	49.5 ± 14.6	46.6 ± 12.8	t=0.722	0.475
Symptom Duration (months), mean ± SD	18.4 ± 29.8	15.2 ± 23.7	24.1 ± 38.5	t=1.050	0.298
Sex (male,female=n-male)	34 (64.2)	23 (67.6)	11 (57.9)	χ² =0.504	0.478
Smoking History, n (%)	23 (43.4)	11 (32.4)	12 (63.2)	χ² =4.709	**0.030**
Allergic Rhinitis Comorbidity, n (%)	27(50.9)	18 (52.9)	9 (47.4)	χ² =2.906	0.208
Comorbidities, n (%)	17 (32.1)	10 (29.4)	7 (36.8)	χ² =0.309	0.578
Previous Surgery, n (%)	21 (39.6)	6 (17.6)	15 (78.9)	χ² =19.145	**0.001**
Affected Nasal Cavity (right), n (%)	30 (56.6)	15 (78.9)	15 (44.1)	χ² =6.109	**0.014**
Lesion Origin Site:the root of the tumor					
Nasal cavity	3 (5.7)	3 (8.8)	0 (0)	χ² =7.039	**0.019**
Ostiomeatal complex (OMC)*	24 (45.3)	19 (55.9)	5 (26.3)		
- Other sites†	26 (49.1)	12 (35.3)	14 (73.7)		
Surgical Approach					
Open sinus surgery via the middle nasal canal	40 (75.5)	28 (82.4)	12 (63.2)	χ² =2.426	0.119
via the middle nasal ,combined with other approaches (Pre-lacrimal recess or supraorbital eyebrow incisions)	13 (24.5)	6 (17.6)	7 (36.8)		
Krouse Staging System					
T1,localized to the nasal cavity	3 (5.7)	3 (8.8)	0 (0)	χ² =3.216	0.198
T2,Involves the ostiomedtal complex and ethmoid sinus	22 (41.5)	16 (47.1)	6 (31.6)		
T3.Invasion of the maxillary sinus walls,sphenoid sinus ,or frontal sinus	28 (52.8)	15 (44.1)	13 (68.4)		
T4,Extension beyond the paranasal sinuses into adjacent structures					

*OMC, Ostiomeatal complex.

†Other sites: Includes sphenoid sinus and multi-region involvement.

‡Combined approaches: Pre-lacrimal recess or supraorbital eyebrow incisions.

### 3.4 Multivariate analysis of factors influencing recurrence post-endoscopic surgery

Factors associated with recurrence identified in univariate analysis were included in the multivariate analysis using logistic regression. The results showed (**Table 3**) that smoking, right nasal cavity involvement, and past surgical history were significant risk factors for tumor recurrence (*P* < 0.05).

**Table 3:** Multivariate logistic regression analysis of factors associated with recurrence of sinonasal inverted papilloma.

Variable	β	SE	Wald χ²	Adjusted OR (95% CI)	P-value
Smoking History	2.311	1.038	4.954	10.08 (1.32—77.15)	0.026
Affected Nasal Cavity (right)	2.052	1.024	4.013	7.79 (1.05—57.99)	0.045
Prior Surgical Intervention	2.849	0.948	9.021	17.26 (2.69—110.76)	0.003

## 4 Discussion

Sinonasal inverted papilloma is a benign tumor occurring in the squamous epithelial mucosa of the nasal cavity and sinuses. SNIP accounts for 0.4% to 4.7% of all nasal tumor cases, with an annual incidence rate of 0.6 to 1.5 cases per 100,000 people (9, 10). The majority of SNIP cases occur in adults, with an average age at diagnosis of 55 years and a male-to-female ratio of 2 to 5:1 (11). The etiology remains unclear but may be related to factors such as smoking, allergies, or occupational exposure (12). Studies indicate that 17% to 42% of cases have associations with human papillomavirus (HPV types 6, 11, 16, 18) and Epstein-Barr(EB) virus (13, 14). In this study, all patients were adults, with an average age of 48 years, and a male-to-female ratio of 1.79:1.

SNIP predominantly manifests with sinusitis-like symptoms (nasal obstruction/headache/purulent discharge), complicating clinical differentiation. Tumor distribution patterns align with established evidence: maxillary sinus (49.1%) > ethmoid (22.6%), consistent with IP’s anatomical predilection. Characteristic endoscopic findings include unilateral pale-red fibrovascular masses (96.2% visibility), often coexisting with inflammatory polyps (23.5% in our cohort). CT reveals pathognomonic bone remodeling (43.4% cystic changes) through hyperostosis/local destruction, with emerging evidence supporting tumor origin localization via neo-osteogenesis patterns (15, 16). Proposed mechanisms involve tumor-induced osteogenic angiogenesis mediated by BMP-2/VEGF crosstalk (17).

MRI provides a direct evaluation of the extent of invasion and can clearly demonstrate the tumor’s origin and growth direction, distinguishing the tumor from associated obstructive inflammation and polyps. The imaging features of inverted papilloma on MRI exhibit convoluted structures resembling brain gyri; as the papilloma grows and inverts toward the base, it forms a serpentine structure on T2-weighted images, with radiating distributions emanating from the origin to the periphery, associated with edema (high signal) and epithelium proliferation (low signal), with notable enhancement upon contrast administration (18).

Surgical treatment remains the primary method for managing SNIP, and endoscopic techniques are the leading approach. A study by Zhang et al. (19) analyzed the effectiveness of endoscopic surgery to treat SNIP both domestically and internationally, revealing recurrence rates of 6.4% to 21.6% overseas and 10.9% to 15.0% domestically. Compared to external incision surgery, endoscopic techniques have developed over the past decades, allowing for precise treatment based on the tumor’s root location. These methods minimize external trauma while ensuring complete tumor removal, thereby becoming the mainstream treatment for inverted papilloma.

The tendency for recurrence is a crucial indicator of surgical effectiveness. Most recurring tumors post-surgery are typically due to incomplete treatment or residual lesions (20). Yet, despite advancements in surgical techniques and technologies, recurrence rates remain high. Literature has reported recurrence rates for inverted papilloma as high as 78% while identifying specific risk factors (21), including tobacco exposure, tumor size, high-grade keratinization, squamous proliferation, increased mitotic activity, HPV positivity, bilateral occurrence, and tumor site (22, 23). In our dataset, the rate of recurrence patients was 35.8%, conversely, 64.15% were newly diagnosed patients. In this study, significant correlations were found between smoking, past surgical history, and tumor site(right nasal cavity) with recurrence of NIP (*P* < 0.05). The Krouse staging system and lesion origin site did not exhibit a clear correlation with recurrence (*P* > 0.05).

Smoking is highlighted as a major risk factor for the occurrence and recurrence of head and neck tumors. Smoking is believed to induce nasal mucosa swelling and chronic inflammation, potentially heightening the risk of postoperative recurrence. Smokers had higher recurrence risk, aligning with VEGF overexpression (MFI=77.54 vs 35.99 in non-smokers) (24). Preoperative smoking cessation programs could reduce recurrence.

This study demonstrated a statistically significant association between right-sided tumor localization and disease recurrence (P < 0.05).After multivariate logistic regression analysis, right-sided involvement remained an independent predictor of recurrence (adjusted OR = 7.09, P = 0.045). Although the underlying mechanism requires elucidation, we hypothesize this lateralization effect might be associated with the 8.3 ± 2.1 mL/min higher blood flow in the right carotid artery of right-handed individuals (25), a speculation that warrants confirmation through prospective perfusion imaging studies.

Prior surgical history significantly predicts tumor recurrence (P<0.05), with 78.9% (15/19) of recurrent cases having previous interventions versus 17.6% (6/34) in non-recurrent controls. Recurrence risk escalates with repeated procedures (primary:12%, secondary:27.3-50%), likely through dual mechanisms: structural destabilization of nasal architecture, and immune microenvironment alteration (TGF-β upregulation post-scarring) (26, 27).

Consequently, these factors directly influence tumor recurrence, warranting thorough assessment and management of patients in clinical practice to reduce recurrence risks. However, our study lacks in-depth exploration of the underlying mechanisms influencing these factors and calls for further investigation and discussion. This study has several limitations, most notably the relatively small sample size (n=53), which may reduce statistical power and increase susceptibility to sampling bias, potentially limiting the generalizability of findings. The insignificant Krouse stage-recurrence association (P=0.198) is different from previous studies, possibly due to our limited T1 cases (n=3). Larger cohorts are needed to clarify staging’s prognostic role.

## 5 Conclusion

This study establishes smoking, surgical history, and lesion laterality as key SNIP recurrence predictors. Integrating these factors into preoperative checklists enables personalized surveillance protocols. Future research should explore HPV status and molecular biomarkers (e.g., SCCA) for enhanced risk stratification.

## Data Availability

The original contributions presented in the study are included in the article/supplementary material. Further inquiries can be directed to the corresponding author/.
